# The International Conference on Intelligent Biology and Medicine (ICIBM) 2020: Scalable techniques and algorithms for computational genomics

**DOI:** 10.1186/s12864-020-07256-9

**Published:** 2020-12-29

**Authors:** Wei Zhang, Zhongming Zhao, Kai Wang, Li Shen, Xinghua Shi

**Affiliations:** 1 Department of Computer Science, College of Engineering and Computer Science, University of Central Florida, Orlando, FL 32816 USA; 2 Center for Precision Health, School of Biomedical Informatics, The University of Texas Health Science Center at Houston, Houston, TX 77030 USA; 3 Raymond G. Perelman Center for Cellular and Molecular Therapeutics, Children’s Hospital of Philadelphia, Philadelphia, PA 19104 USA; 4 Department of Pathology and Laboratory Medicine, University of Pennsylvania, Philadelphia, PA 19104 USA; 5 Department of Biostatistics, Epidemiology and Informatics, Perelman School of Medicine, University of Pennsylvania, Philadelphia, PA 19104 USA; 6 Department of Computer and Information Sciences, College of Science and Technology, Temple University, Philadelphia, PA 19122 USA

## Abstract

In this introduction article, we summarize the 2020 International Conference on Intelligent Biology and Medicine (ICIBM 2020) conference which was held on August 9–10, 2020 (virtual conference). We then briefly describe the nine research articles included in this supplement issue. ICIBM 2020 hosted four scientific sections covering current topics in bioinformatics, computational biology, genomics, biomedical informatics, among others. A total of 75 original manuscripts were submitted to ICIBM 2020. All the papers were under rigorous review (at least three reviewers), and highly ranked manuscripts were selected for oral presentation and supplement issues. This genomics supplement issue included nine manuscripts. These articles cover methods and applications for single cell RNA sequencing, multi-omics data integration for gene regulation, gene fusion detection from long-read RNA sequencing, gene co-expression analysis of metabolic pathways in cancer, integrative genome-wide association studies (GWAS) of subcortical imaging phenotype in Alzheimer’s disease, as well as deep learning methods for protein structure prediction, metabolic pathway membership inference, and horizontal gene transfer (HGT) insertion sites prediction.

## Introduction

The 2020 International Conference on Intelligent Biology and Medicine (ICIBM 2020), the official conference of the International Association for Intelligent Biology and Medicine (IAIBM), was successfully held virtually from August 9th to 10th, 2020 due to the coronavirus 2019 (COVID-19) outbreak. Since its inception in 2012, the goal of the ICIBM conference was to provide a forum that fosters interdisciplinary discussion, educational opportunities, and collaborative efforts among the fields of bioinformatics, systems biology, intelligent computing, and medical informatics, which are continuing to evolve at a rapid pace and having a significant impact in biomedical research and innovations.

Building upon the successes of the conferences in the previous years [1–7], ICIBM 2020 turned out to be the largest of all the ICIBM series based on the number of registrations and attendees, thanks to the online platform and free registration. A total of 300 scientists or trainees registered and 291 of them attended. They came from across the world with diverse backgrounds and training ranging from biology, medicine, computer science, bioengineering, bioinformatics, statistics, mathematics, and genomics. We received 75 original manuscripts. These manuscripts covered research topics including next-generation sequencing, single cell analyses, deep learning, genomics and other omics research, system biology, medical applications, computational methods, and novel applications of computational tools. Each paper was carefully reviewed by the program committee members. Among these submissions, we observed more papers coming from emerging fields such as deep learning, new genomic technologies, and big medical data science.

Below, we briefly summarize the scientific program of the ICIBM 2020 and provide an introduction to the nine research articles selected for this BMC Genomics supplement issue.

## Overall scientific program of ICIBM 2020

### Scientific sessions

ICIBM 2020 included four scientific sessions. Speakers in the regular sessions were chosen from those top ranked manuscripts after peer review. The topics in these sessions covered bioinformatics, genomics, systems biology, intelligent computing, data sciences, computational drug discovery, and biomedical informatics. The details of these sessions, including session chairs, authors, presenters, and the title and abstract of each talk were included on the conference website. The four session are:

- Session I: Computational Genomics
- Session II: Biomedical Informatics
- Session III: Genomics and Beyond
- Session IV: Bioinformatics

Here, we provide an editorial report of the supplements to BMC Genomics that include nine research papers selected from 75 manuscripts submitted to ICIBM 2020. Each manuscript was reviewed for two rounds. The first round of review was carried out by at least three reviewers and the manuscripts were substantially revised according to reviewers’ critiques. The revision was further reviewed by at least two reviewers (most by three reviewers) before being accepted into the supplement issue. Other selected papers were accepted in other BMC journals: BMC Bioinformatics, BMC Medical Genomics, and BMC Medical Informatics and Decision Making.

### Summaries of manuscripts in this issue

Below, we summarize the contribution of nine papers included in this supplement issue. These papers cover a wide spectrum of topics in bioinformatics and computational biology, and provide scalable techniques and algorithms for computational genomics.

In the first paper, Cornish et al. [8] proposed a novel method, Red Panda, to accurately identify single nucleotide variations or micro (1-50 bp) insertions and deletions in single-cell RNA sequencing (scRNA-seq) data. Red Panda employs the fact that transcripts represented by scRNA-seq reads necessarily only originate from the chromosomes present in a single cell to decide what is and is not a heterozygous variant. The method utilizes this unique information found in scRNA-seq to achieve better performance compared to methods designed for bulk RNA sequencing.

Multi-omics data integration has now become a routine technique to understand the mechanisms of gene regulation. Cao et al. [9] introduced an R package, *intePareto*, for the integrative analysis of RNA-seq and ChIP-seq data for different histone modifications. Specifically, the method can examine the abundance correlations of histone modification and gene expression. Then, it prioritizes genes with congruent changes in RNA-seq and ChIP-seq between two experimental conditions using Pareto optimization. The results show that integration of RNA-seq data and ChIP-seq data by Pareto optimization outperformed an unsupervised method based on Bayesian inference of a hierarchical model and a method using single omics data only.

The paper by Gao et al. [10] applied scRNA-seq to profile human and mouse bone marrow cells, and inferred transcriptome regulatory networks in each species to characterize transcriptional programs controlling hematopoietic stem cell differentiation. A network reconstruction algorithm was designed to conduct comparative transcriptomic analysis of hematopoietic gene co-expression and transcription regulation in bone marrow cells in different species. The results show that the co-expression network connectivity of hematopoiesis-related genes is well conserved between mouse and human.

Long-read RNA-seq techniques are able to sequence full-length transcripts, so they are expected to overcome inherited limitations of short-read RNA-seq techniques. Liu et al. [11] introduced a fast computational tool, LongGF, to efficiently detect candidate gene fusion events from long-read RNA-seq data. The pipeline takes aligned BAM files as input and outputs a prioritized list of candidate gene fusions together with their supporting long reads. LongGF was evaluated on a Nanopore sequencing dataset and a PacBio sequencing dataset and the results showed that LongGF achieved better performance to detect known gene fusions over current computational methods.

Horizontal Gene Transfer (HGT) is generally defined as exchange of genetic materials between distant species that are not in a parent-offspring relationship. The HGT insertion sites are important to study the HGT mechanisms. Li et al. [12] introduced a deep residual network, DeepHGT, to predict HGT insertion sites on genomes based on the sequence pattern. By applying DeepHGT on bacteria genomes and their coding genes, the model can estimate the likelihood of bacteria genomes harboring HGT insertions sites and detect bacterial genes enriched with potential HGT insertions sites. The preliminary results show that DeepHGT outperformed existing machine learning models on a large sequence dataset from 262 metagenomic samples.

The paper by Zhang et al. [13] studied the relation between metabolic pathway gene expression and cancer prognosis by recently established bioinformatics methods. First, the study evaluated whether the expression of genes in the metabolic pathway have any predictability for cancer outcome and the results showed that the higher expression corresponds to worse survival. Second, this study also explored the co-expression patterns within the metabolic pathways and differential expression of the metabolic pathways between paired tumor and normal tissues. The analyses demonstrated the disruption of metabolic pathway gene expression between tumor and normal samples.

The paper by Cartealy et al. [14] proposed a neural network based method to infer protein’s membership in metabolic pathways using both gene ontology similarity and sequential features between a query protein and proteins that are known to the members of a particular metabolic pathway. The method was designed with a network architecture tailored to facilitate the integration of features from different sources. The results demonstrated that the neural network based method outperformed other canonical classification models (i.e., support vector machine and random forest) and the methods that are specifically designed to use the gene ontology features alone.

Genome-wide association studies (GWAS) can detect variants/genes associated with brain imaging quantitative traits (QTs) in Alzheimer’s disease (AD). Meng et al. [15] applied multivariate gene-based association test (MGAS) to perform GWAS on eight AD-relevant subcortical imaging measures by exploring single-SNP-multi-QT associations. Then the identified genes were mapped to the protein-protein interaction (PPI) network to detect enriched consensus modules. The statistical power of coupling MGAS with network analysis was higher than traditional GWAS methods and generated new findings that were missed by GWAS. The analysis identified several genes associated with the studied imaging phenotypes and detected five consensus modules from the PPI network which provide novel insights into the molecular mechanism of AD.

To understand the biological function of proteins, it is important to accurately predict protein structure. Zhang et al. [16] proposed a new template-based modelling method, ThreaderAI, to improve protein tertiary structure prediction. The method formulates the prediction task of aligning query sequence with template as the canonical pixel classification problem in computer vision and applied deep residual neural network in prediction. Experimental results showed that the ThreaderAI can significantly improve the prediction accuracy compared to popular template-based modelling methods, especially on the proteins that do not have close homologs with known structures.

## Conference organization

### 2020 International conference on intelligent biology and medicine (ICIBM 2020)

(August 9–10, 2020, Virtual Conference)

We would like to express our sincere gratitude to the general chairs and the members of the steering, program, publication, publicity, award and trainee committees, as well as to all the reviewers, who spent their valuable time and effort on making ICIBM 2020 a success. The conference accomplishments are the results of support and hard work of all these people during the COVID-19 pandemic.

**Host:** The International Association for Intelligent Biology and Medicine (IAIBM).

**General Chairs** Kai Wang (Children’s Hospital of Philadelphia and University of Pennsylvania) and Zhongming Zhao (The University of Texas Health Science Center at Houston).

**Steering Committee** Yidong Chen (The University of Texas Health Science Center at San Antonio), Xiaohua Tony Hu (Drexel University), Kun Huang (Indiana University), Sudhir Kumar (Temple University), Jason Moore (University of Pennsylvania), Yi Xing (University of Pennsylvania).

**Program Committee** Co**-**Chairs: Xinghua (Mindy) Shi (Temple University), Li Shen (University of Pennsylvania). Members: Yongsheng Bai (University of Michigan) Xiao Chang (Children’s Hospital of Philadelphia), Bin Chen (Michigan State University), Li Chen (Indiana University School of Medicine), Yidong Chen (The University of Texas Health Science Center at San Antonio), Jianlin Cheng (University of Missouri Columbia), Lijun Cheng (Ohio State University), Zechen Chong (The University of Alabama at Birmingham), Yulin Dai (University of Texas Health Science Center at Houston), Yang Ding (Beijing Institute of Radiation Medicine, China), Abolfazl Doostparast (University of California, Santa Barbara), Lana Garmire (University of Michigan), Jun-Tao Guo (University of North Carolina at Charlotte), Yan Guo (University of New Mexico), Zhi Han (Indiana University School of Medicine), Matthew Hayes (Xavier University of Louisiana), Zhe He (Florida State University), Ting Hu (Queen’s University, Canada), Heng Huang (University of Pittsburgh & JD Digits), Kun Huang (Indiana University School of Medicine), Tao Huang (Chinese Academy of Sciences, China), Weichun Huang (National Institutes of Health), Yufei Huang (University of Texas at San Antonio), Zhi-Liang Ji (Xiamen University, China), Peilin Jia (The University of Texas Health Science Center at Houston), Victor Jin (The University of Texas Medical School at San Antonio), Yufang Jin (University of Texas at San Antonio), Travis Johnson (The Ohio State University), Dokyoon Kim (University of Pennsylvania), Rui Kuang (University of Minnesota Twin Cities), Aimin Li (Xi’an University of Technology, China), Fuhai Li (Washington University School of Medicine) Jia Li (Texas A&M University), Tao Li (Nankai University, China), Li Liao (University of Delaware), Honghuang Lin (Boston University School of Medicine), Ke Liu (Michigan State University), Xiaoming Liu (University of South Florida), Yaping Liu (Cincinnati Children’s Hospital Medical Center), Zhandong Liu (Baylor College of Medicine), Shuang Luan (University of New Mexico), Qin Ma (The Ohio State University), Maciej Pietrzak (The Ohio State University), Mirjana Maletic-Savatic (Baylor College of Medicine), Patricio Manque (Universidad Mayor, Chile), Huaiyu Mi (University of Southern California), Nitish Mishra (University of Nebraska Medical Center), Tabrez Anwar Shamim Mohammad (Greehey Children’s Cancer Research Institute), Zhengqing Ouyang (University of Massachusetts, Amherst), Hatice Gulcin Ozer (The Ohio State University), Ranadip Pal (Texas Tech University), Jiang Qian (Johns Hopkins University), Guimin Qin (Xidian University, China), Boshu Ru (Merck & Co. Inc), Jianhua Ruan (The University of Texas at San Antonio), Wei Shao (Indiana University Bloomington), Yang Shen (Texas A&M University), Xiaofeng Song (Nanjing University of Aeronautics & Astronautics, China), Fengzhu Sun (University of Southern California), Zhifu Sun (Mayo Clinic), Wing-Kin Sung (National University of Singapore, Singapore), Manabu Torii (Kaiser Permanente), Fuchiang Rich Tsui (Children’s Hospital of Philadelphia and University of Pennsylvania), Jun Wan (Indiana University School of Medicine), Jiandong Wang (University of South Carolina), Jiayin Wang (Xi’an Jiaotong University, China), Junbai Wang (Radium Hospital, Norway), Kai Wang (Children’s Hospital of Philadelphia), Yufeng Wang (University of Texas at San Antonio), Chaochun Wei (Shanghai Jiao Tong University, China), Lei Wei (Roswell Park Comprehensive Cancer Center), Yingying Wei (The Chinese University of Hong Kong, Hong Kong), Jia Wen (University of North Carolina at Chapel Hill), Chunhua Weng (Columbia University), Huanmei Wu (Indiana University - Purdue University Indianapolis), Yonghui Wu (University of Florida), Junfeng Xia (Anhui University, China), Lei Xie (City University of New York), Hua Xu (The University of Texas School of Biomedical Informatics at Houston), Min Xu (Carnegie Mellon University), Jianhua Xuan (Virginia Tech), Yu Xue (Huazhong University of Science and Technology, China), Jingwen Yan (Indiana University - Purdue University Indianapolis), Sungroh, Yoon (Seoul National University, Korea), Liang Zhan (University of Pittsburgh), Chi Zhang (Indiana University School of Medicine), Han Zhang (Nankai University, China), Rui Zhang (Michigan Tech), Shaojie Zhang (University of Central Florida), Wei Zhang (University of Central Florida), Ying Zhang (University of Rhode Island), Zuoyi Zhang (Indiana University), Zhongming Zhao (The University of Texas Health Science Center at Houston), Jim Zheng (The University of Texas Health Science Center at Houston), Wanding Zhou (Children’s Hospital of Philadelphia), Yunyun Zhou (University of Mississippi), Dajiang Zhu (University of Texas at Arlington).

**Publication Committee** Co-Chairs: Yan Guo (University of New Mexico), Wei Zhang (University of Central Florida).

**Workshop/Tutorial Committee** Chair: Yulin Dai (University of Texas Health Science Center at Houston).

**Publicity Committee** Co**-**Chairs: Yu Xue (Huazhong University of Science and Technology, China), Kwangsik Nho (Indiana University).

**Award Committee** Chair: Jinchuan Xing (Rutgers University).

**Trainee Committee** Chair: James Havrilla (Children’s Hospital of Philadelphia).

**Local Organization Committee** Co-Chairs: Dokyoon Kim (University of Pennsylvania), Wanding Zhou (Children’s Hospital of Philadelphia).
